# Microbes and hydrothermal environments: An annotated selection of World Wide Web sites relevant to the topics in environmental microbiology

**DOI:** 10.1111/1758-2229.13141

**Published:** 2023-01-19

**Authors:** Lawrence P. Wackett

**Affiliations:** ^1^ McKnight Professor, Department of Biochemistry, Molecular Biology & Biophysics BioTechnology Institute, University of Minnesota, St. Paul, MN 55108, USA

## Microbes that keep hydrothermal vents pumping

### https://ocean.si.edu/ecosystems/deep‐sea/microbes‐keep‐hydrothermal‐vents‐pumping

This article written by Smithsonian Ocean staff provides a good overview of hydrothermal vent communities suitable for non‐scientific readers.

## Microbes and minerals in deep‐sea environments

### https://www.mdpi.com/2075‐163X/11/12/1324

This report reviews the microbial diversity and microbe‐mineral interactions in deep‐ocean hydrothermal environments.

## Core communities at hydrothermal vents

### https://www.nature.com/articles/s43705‐021‐00031‐1

This report dealt with the rich taxonomic diversity in vent communities, with a particular focus on those within the tubeworm *Ridgeia piscesae*.

## Microbial succession at vent sulfide chimneys

### https://microbiomejournal.biomedcentral.com/articles/10.1186/s40168‐020‐00851‐8

This article covered microbial succession during a change from active to inactive deep‐sea sulfide chimneys.

## Microbiomes near hydrothermal vents

### https://www.nature.com/articles/s41579‐019‐0160‐2

This paper is a broad overview of microbial population at deep‐sea areas containing hydrothermal vents.

## Gene movement at hydrothermal vents

### https://astrobiology.nasa.gov/news/the‐gain‐and‐loss‐of‐genes‐at‐hydrothermal‐vents/

This page provides a summary of a published paper on gene transfer in deep‐sea hydrothermal vent bacteria. It contains a link to the original article.

## Geomicrobiology of deep‐sea thermal vents

### https://www.science.org/doi/10.1126/science.229.4715.717

This article by Jannasch and Mottl is one of the classics in the field of hydrothermal vent chemistry and microbiology.

## Chemosynthetic oases: YouTube

### https://www.youtube.com/watch?v=1LrcTa0dDmw

There are numerous videos on life in deep‐sea environments. This is one of the longest and most comprehensive of those.

## Deep sea vents

### https://microbewiki.kenyon.edu/index.php/Deep_sea_vent

This page of the Microbe Wiki describes deep sea vent properties, global locations, bacterial communities, and eukaryotic organisms present in those environments.

## Obligately photosynthetic bacterium from a deep‐sea vent

### https://www.pnas.org/doi/10.1073/pnas.0503674102

This report describes the isolation and characterization of a green sulfur bacterial species from a deep‐ocean hydrothermal vent that is proposed to use geothermal radiation as a source of photons to be absorbed by its photosynthetic pigments.

## Hydrogen cycling in deep‐ocean hydrothermal ecosystems

### https://www.frontiersin.org/articles/10.3389/fmicb.2018.02873/full

This article reviews knowledge of, and techniques for studying, hydrogen uptake and hydrogen evolving enzymes involved in hydrogen cycling at hydrothermal vents.

## Viruses in hydrothermal vents

### https://www.ncbi.nlm.nih.gov/pmc/articles/PMC8269205/

This study samples several deep‐sea hydrothermal vents to sequence viruses that were present. The general conclusions were that diverse viruses were present with different assemblages that are highly localized to specific areas and hosts.

